# Morbific Infection*Read before the Louisville Medico-Chirurgical Society, March 11, 1898.

**Published:** 1898-05

**Authors:** Frank C. Wilson

**Affiliations:** Professor of Diseases of the Chest and Physical Diagnosis in the Hospital College of Medicine; President of the Louisville Medico-Chirurgical Society, etc., Louisville, Ky.


### MORBIFIC INFECTION.*

By FRANK C. WILSON, M.D.

Professor of Diseases of the Chest and Physical Diagnosis in the Hospital College of Medicine; President of the Louisville Medico-Chirurgical Society, etc., Louisville, Ky

That diseases are communicated from patient to patient has been known from ancient times, but few of us realize how numerous are the avenues through which infectious germs gain access to the system. All admit the contagiousness of such diseases as smallpox, scarlet fever, measles, whooping cough and mumps, but only in recent years has the communicability of tuberculosis been admitted. In my limited experience I can recall scores of instances where husbands or wives, strong and healthy, with no inherited tendencies to tuberculosis, have developed the disease within a few months after burying a companion. Every contagious or infectious disease has its specific contagium. Whether this be a specific germ or an impalpable and unrecognizable poison is immaterial; it is capable of self-multiplication and producing the disease in another system.

The microscope in the hands of the bacteriologist has accomplished much in discovering the specific germ in many diseases, and no doubt, sooner or later, no exceptions will be found. In some instances the germ or morbific material passes directly from one system to another, as in smallpox, measles, scarlet fever, etc. In other instances the contagium must first multiply in a suitable soil, under suitable conditions and surroundings, before being able to reproduce the disease in other systems. This is the case in typhoid fever, yellow fever and cholera.

The history of the yellow fever epidemic in this city in 1878 clearly proved the facts. Large numbers of cases were brought here from the South, were nursed and treated in the fever hospital; not a single physician or nurse contracted the disease from them. The temperature and conditions were not such as to favor the propagation and multiplication of the poison to such a degree as to infect others brought within its influence. In the neighborhood of the Nashville depot all the necessary conditions did exist in the little baggage-room at the end of the platform, which was used as a store-room for soiled clothing and baggage and blankets taken from the trains bringing in the fever cases. This house was covered with a tin roof, had one window and a door, and was closed most of the time. With the hot sun beating upon the roof the temperature was continually at or above a hundred degrees. The germs were present, filth abounded, and temperature was maintained as in an incubator. From this as a focal center the poison gradually extended, attacking first the two ticket-agents, fifty yards in one direction, then several cases across the street in another direction. In this way twenty-

*Read before the Louisville Medico-Chirurgical Society, March 11, 1898.

five or thirty cases developed, which were just as genuine cases of yellow fever as those brought from the South. Just as soon as the source of the development of contagium was recognized and broken up the cases ceased to develop. The conditions existing in the little baggage-room, the filthy soil, the implanted germs and the incubating temperature were just such as were found in the South, and gave rise to the disease just as it does in the South. If the continuous high temperature is lacking, or the filthy soil is not present, though the germs be numerous, they will not develop nor give rise to an epidemic. The germs or contagium contained in sputum, fecal and urinary secretions, or crusts and scales from the surface conveying the disease, may gain access through the respiratory tract, through the intestinal tract, or by absorption through an abrasion of the surface.

The sputum for a consumptive patient swarms with bacilli in immense numbers. It has been estimated that the expectoration from one well-develped case of pulmonary tuberculosis will contain several millions of bacilli, enough to inoculate every person in this city. This sputum, if allowed to dry, becomes pulverized into a fine dusty powder, so small that each particle containing one or more germs will be caught up by the currents of air and float about in the atmosphere until drawn into the respiratory passages of some luckless victim. Lodging upon the mucous membrane, it forms a colony by self-multiplication, gradually encroaching upon the surrounding tissues, and every now and then implanting new colonies in the immediate neighborhood, involving more and more of the structure, or, by breaking into the blood or lymph channels, distributing germs widely throughout the entire system, resulting in general tuberculosis.

The sputum, instead of being allowed to dry, may be thrown into the vault, and after a time, when this is cleaned, may find its way to the country fields, lodge upon the grass and be devoured by the cows grazing in the meadows. In the system of the beautiful and gentle Jersey cow is a suitable soil for its development, and the milk may carry the germs back to the city to be taken into the stomach of some bottle-fed baby or some lover of milk. Intestinal tuberculosis may be the result. We know that tuberculosis sometimes affects domestic fowls. I have myself seen a consumptive, walking in a barnyard, expectorate upon the ground, the sputum being eagerly devoured by the chickens gathered around it. Who can say that tuberculosis developed in the bodies of these fowls, if they be served as spring chickens at the table, may not carry the germs into the systems of those feasting upon them.

The kiss of the consumptive patient impressed upon the lips of the dearly loved one may carry with it the deadly bacillus, as fatal as a draught from a poisoned chalice. No less destructive may be the kiss of a syphilitic subject having a mucous patch upon the lip. I have known an innocent child, carried in the arms of a nurse, infected in this way, the result being a chancre upon the tongue, which, in turn, inoculated the nipple of the nursing mother. Both mother and child developed constitutional symptoms. All this was traceable to the poisoned kiss of a friend of the nurse. Even the communion cup is not free from the charge of conveying poisonous germs, and one diseased lip may in this way infect scores who sip from the same vessel. To avoid this danger the custom of using small individual cups, however inconvenient, is fast gaining ground, and when thoroughly sterilized before each use safety is assured. Even the apparently innocent custom of shaking hands may convey germs of disease or parasites from one to the other, the communication of scabies being a familiar example.

Contamination of the water supply is a fruitful source of of disease. The contagium of typhoid fever, of cholera, and, in fact, of any infectious disease, may be thus introduced into the system. I well remember, when in attendance upon an epidemic of cholera in Lancaster, Ky., some years ago, tracing very clearly the outbreak to the contamination of a well at the foot of the hillside where the discharges from the first case, a refugee from Nashville, were thrown out and washed down by the rain. All the succeeding cases developed among the families supplied by that well.

Rigid sanitary and quarantine regulations are adopted against epidemic diseases, such as yellow fever, cholera, smallpox, scarlet fever and diphtheria, and yet the disease that destroys one-seventh of the human race is allowed to stalk abroad in the land almost without let or hindrance. When we reflect that each case scatters abroad germs enough to inoculate several hundred thousand persons every day, it is a marvel that any one escapes. Not until the public and each individual subject realize fully the danger and adopt stringent measures looking to the thorough destruction by fire or by other potent disinfectant of every morbific material will safety be secured against the spread of contagious and infectious diseases.—*American Practitioner and News.*

